# Reallocating childhood physical activity and sedentary time and adolescent mental health: a compositional data analysis of the UK Millennium Cohort Study

**DOI:** 10.3389/fpsyg.2026.1932607

**Published:** 2026-09-28

**Authors:** Yucheng Zhu, Yingguo Hu, Yuhong Zhu

**Affiliations:** 1 School of Physical Education, Yunnan Minzu University, Kunming, China; 2 School of Physical Education, Yunnan Normal University, Kunming, China

**Keywords:** compositional data analysis, isotemporal substitution, mental health, physical activity, sedentary behavior

## Abstract

**Background:**

Physical activity during childhood may promote mental health, but the effects of reallocating time between intensity zones on specific mental health dimensions remain poorly understood. To address this research gap, this study examined prospective associations between the composition of device-based physical activity at age 7 and multiple mental health outcomes at age 14 using a compositional data analysis (CoDA) framework that accounts for the constrained nature of daily time use.

**Methods:**

Data were drawn from 5,145 children (48.1% boys) in the UK Millennium Cohort Study. Physical activity was measured using waist-worn ActiGraph GT1M accelerometers at age 7 and classified into sedentary behavior (SB), light-intensity physical activity (LPA), and moderate-to-vigorous-intensity physical activity (MVPA). Mental health was assessed at age 14 using the parent-reported Strengths and Difficulties Questionnaire (SDQ). Isometric log-ratio (ILR) transformed activity compositions were entered into linear regression models adjusting for baseline SDQ at age 7 and sociodemographic covariates. Compositional isotemporal substitution models estimated the predicted change in each SDQ outcome associated with reallocating 15 min per day between activity types. All analyses were stratified by sex. Conventional linear regression was conducted as a sensitivity analysis.

**Results:**

Reallocating 15 min per day from LPA to MVPA was associated with fewer peer problems (delta = −0.050, *p* = 0.008). However, reallocating time from SB to MVPA was concurrently associated with higher hyperactivity (delta = 0.050, *p* = 0.002) and externalizing scores. Sex-stratified analyses revealed that the associations with peer problems were more pronounced among boys (e.g., LPA to MVPA on peer problems: delta = −0.097, *p* < 0.001) and were absent in girls, a pattern supported by formal interaction testing (*p* = 0.004). Conventional regression yielded directionally consistent results.

**Conclusion:**

Childhood physical activity composition is prospectively associated with adolescent mental health in a dimension-specific manner, with suggestive evidence of sex differences. Modeled reallocation of time to MVPA was associated with more favorable peer-relationship and internalizing outcomes, particularly in boys, while associations with externalizing outcomes likely reflect reverse causality. These findings highlight the potential relevance of physical activity intensity for future longitudinal and intervention research on youth mental health.

## 1 Introduction

Mental health problems among children and adolescents have become a major public health concern globally. The World Health Organization estimates that approximately one in seven adolescents aged 10–19 years is affected by a mental disorder, accounting for roughly 15% of the global disease burden in this age group (World Health Organization, 2021). Internalizing difficulties such as emotional distress and peer relationship problems, as well as externalizing difficulties including conduct and hyperactivity symptoms, tend to increase during the transition from middle childhood to adolescence (Sellers et al., 2019). At the same time, physical inactivity is widespread among young people. Global surveillance data show that approximately 80% of adolescents do not meet the recommended 60 min per day of moderate-to-vigorous-intensity physical activity (MVPA) (Bull et al., 2020; Guthold et al., 2020). Physical activity is defined as any bodily movement produced by skeletal muscles that results in energy expenditure (Caspersen et al., 1985). Sedentary behavior (SB) is defined as any waking behavior characterized by an energy expenditure of 1.5 metabolic equivalents or less while in a sitting, reclining, or lying posture (Tremblay et al., 2017). Given the high co-occurrence of insufficient physical activity and rising mental health problems, elucidating dose-response relationships between physical activity and mental health outcomes is warranted. Among the parameters that determine the physical activity dose, intensity is particularly relevant because it differentiates behaviors that may have distinct physiological and psychosocial effects (Hale et al., 2023).

A growing body of evidence supports a positive association between physical activity and youth mental health. In a systematic review and meta-analysis encompassing studies of preschoolers through adolescents, Rodriguez-Ayllon et al. (2019) reported that higher physical activity levels were generally associated with more favorable mental health outcomes, though heterogeneity across study designs, measurement tools, and outcome definitions limited the strength of conclusions. Biddle et al. 2019) reached a similar overall assessment, while noting that the evidence base remained insufficient to establish causal direction, identify dose-response gradients, or determine whether MVPA conferred stronger benefits than light-intensity physical activity (LPA). Two aspects of this evidence gap deserve particular attention. First, most studies have relied on cross-sectional designs or short follow-up periods, limiting the ability to capture how childhood activity patterns shape mental health trajectories into adolescence. Second, the question of intensity specificity remains largely unresolved: few longitudinal studies have simultaneously examined the independent and relative contributions of SB, LPA, and MVPA to multiple dimensions of mental health (Alosaini et al., 2023). Notably, SB has been identified as a distinct risk factor for mental health in young people, independent of physical activity levels (Rodriguez-Ayllon et al., 2019).

A key methodological challenge in addressing these questions lies in the compositional nature of daily time use. The minutes a child spends in SB, LPA, and MVPA during waking hours must sum to total wear time, creating an inherent constraint among these activity categories. When entered as separate predictors in a conventional regression model, these components violate the assumption of statistical independence, give rise to multicollinearity, and yield coefficients whose interpretation is ambiguous because the reference behavior (i.e., what decreases when the target behavior increases) is not specified (Chastin et al., 2015; Dumuid et al., 2018). Compositional data analysis (CoDA) provides a statistically appropriate framework for handling such constrained data. Through an isometric log-ratio (ILR) transformation, CoDA converts the activity composition into a set of unconstrained coordinates suitable for standard regression modeling. When further combined with the compositional isotemporal substitution model (Dumuid et al., 2019), CoDA enables estimation of the expected change in a health outcome when a fixed duration of time is reallocated from one behavior to another while holding the remaining behaviors constant. This approach translates statistical associations into operationally meaningful scenarios, for instance, quantifying what might be expected if a child replaced 15 min of daily SB with MVPA. Although CoDA has gained traction in physical activity epidemiology in recent years (Fairclough et al., 2021; Verswijveren et al., 2022), its application to longitudinal data linking childhood activity compositions with adolescent mental health remains limited. Other analytical approaches, including machine learning methods, have also been applied to examine associations between movement behaviors and mental health (Pan et al., 2026a), offering complementary perspectives.

Several studies have used data from the UK Millennium Cohort Study (MCS) to investigate prospective associations between objectively measured physical activity and mental health in children. Ahn et al. (2018) found that each additional 15 minutes per day of MVPA at age 7 was associated with a reduction of 0.077 points in peer problems at age 11, with stronger effects observed in boys. Werneck et al. (2025) extended the MCS follow-up through ages 11, 14, and 17, confirming that childhood MVPA predicted lower peer problems yet higher hyperactivity scores among boys. Li et al. (2025) reported protective associations between MVPA and internalizing problems in a subsample of overweight and obese MCS participants (*n* = 858), with effect sizes increasing at the age 14 follow-up. Wang and Wang (2026) observed that MVPA was associated with reduced emotional and peer difficulties in the full MCS sample, though their analyses were limited to these two SDQ subscales. Additional MCS-based studies have examined related questions, including longitudinal associations between physical activity-sedentary time balance and internalizing/externalizing problems (Cai et al., 2026), prospective links between physical activity and behavioral outcomes in preterm youth (Cheng et al., 2024), SB and behavioral problems in children with overweight/obesity (Guan et al., 2025), and the timing of MVPA in relation to psychosocial outcomes (Pan et al., 2026b). Despite this accumulating evidence, all prior studies employed conventional regression models without accounting for the compositional structure of activity time. None applied isotemporal substitution within a CoDA framework, and the exploration of sex differences has been inconsistent across studies: some controlled for sex without stratification, others stratified but did not examine the full range of mental health dimensions (Del Pozo-Cruz et al., 2018; Dumuid et al., 2019).

The present study aimed to address these limitations using data from the MCS, a large nationally designed birth cohort. We applied the CoDA framework and the compositional isotemporal substitution model to accelerometer-measured physical activity at age 7 to examine prospective associations with multiple SDQ outcomes at age 14, spanning a 7-year follow-up from middle childhood into early adolescence. Our analyses had two main objectives. The first was to examine whether the relative proportion of MVPA vs. LPA in children's daily activity composition was independently associated with subsequent mental health outcomes, as captured by the ILR coordinates from the CoDA regression. The second was to estimate the expected change in each SDQ dimension when 15 min per day were reallocated from SB or LPA to MVPA, and to test whether these substitution effects differed between boys and girls. All models were stratified by sex, motivated by converging evidence that MVPA may be more strongly associated with mental health benefits in boys than in girls (Ahn et al., 2018; Werneck et al., 2025). To assess the robustness of our findings, we also conducted conventional linear regression as a sensitivity analysis, allowing direct comparison with the existing MCS literature that has relied on this approach.

## 2 Methods

### 2.1 Study design and data source

This study employed a prospective cohort design using data from the UK Millennium Cohort Study (MCS). The MCS is an ongoing nationally representative birth cohort study that has followed approximately 19,000 children born between 2000 and 2002 in England, Scotland, Wales, and Northern Ireland (Connelly and Platt, 2014). The initial sample was drawn using a stratified, multistage clustered probability design based on the UK Child Benefit records, with deliberate oversampling of families in areas with high proportions of ethnic minorities, economically disadvantaged communities, and the three smaller UK nations to ensure adequate representation across demographic groups (Plewis, 2007).

Data from two MCS sweeps were used: Sweep 4 (age 7, conducted in 2008) and Sweep 6 (age 14, conducted in 2015). Sweep 5 (age 11, conducted in 2012) was not used in the present analyses. Accelerometer-measured physical activity at Sweep 4 served as the exposure variable, while the Strengths and Difficulties Questionnaire (SDQ) administered at Sweep 6 provided the primary outcome measures. SDQ data from Sweep 4 were used to adjust for baseline mental health status. This design established a 7-year prospective observation window in which the assessment of the exposure preceded the measurement of the outcome.

Ethical approval for each MCS sweep was granted by the relevant National Health Service Research Ethics Committees. Written informed consent was obtained from the primary caregivers of all participating children.

The present study constitutes a secondary analysis of anonymized, publicly available data from the MCS. No additional ethical approval was required for this secondary analysis, as the data were accessed through the UK Data Service under a standard end-user license agreement.

### 2.2 Participants

At Sweep 4, a total of 13,681 children were invited to participate in an accelerometer substudy. Of these, 6,675 provided data classified as reliable according to the criteria described below. For the present analysis, participants were further excluded if they were missing baseline SDQ data at age 7 (*n* = 593), SDQ outcome data at age 14 (*n* = 680), or data on any covariate (*n* = 257). The final analytic sample comprised 5,145 children (2,475 boys and 2,670 girls).

The participant selection process is summarized in Supplementary Figure S2. Of the 13,681 children invited to the accelerometer substudy, 7,006 did not provide valid accelerometer data (i.e., fewer than 2 days with at least 10 h of wear time). Among the 6,675 children with valid accelerometer data, 593 were excluded for missing baseline SDQ data at age 7, 680 for missing SDQ outcome data at age 14, and 257 for missing covariate data.

### 2.3 Physical activity measurement

Physical activity was objectively measured using the ActiGraph GT1M accelerometer (ActiGraph LLC, Pensacola, FL, USA), a uniaxial waist-worn device that records accelerations sampled at 15-s epochs (Rich et al., 2013). Children were instructed to wear the accelerometer during all waking hours for seven consecutive days, removing it only for sleep, bathing, and aquatic activities. Periods of consecutive zero counts lasting 20 min or longer were classified as non-wear time and excluded from the analysis. A valid day was defined as one with at least 10 h of recorded wear time, and a minimum of two valid days was required for inclusion in the dataset (Griffiths et al., 2013).

Recorded activity was classified into four intensity categories using the count-per-minute thresholds established by Pulsford et al. (2011), which were specifically calibrated for 7-year-old children participating in the MCS: sedentary behavior (SB; less than 100 counts per minute), light-intensity physical activity (LPA; 100 to 2,240 counts per minute), moderate-intensity physical activity (MPA; 2,241 to 3,840 counts per minute), and vigorous-intensity physical activity (VPA; greater than 3,840 counts per minute). For the compositional data analysis, MPA and VPA were combined into a single MVPA category, yielding a three-part activity composition (SB, LPA, MVPA). This simplification from four to three components was adopted to improve the interpretability of the isometric log-ratio coordinates and to increase statistical power, consistent with the approach used in comparable studies (Fairclough et al., 2021). All activity variables were expressed as minutes per day averaged across valid days.

### 2.4 Mental health assessment

Mental health was assessed using the parent-reported version of the Strengths and Difficulties Questionnaire (SDQ; Goodman, 1997). The SDQ is a 25-item screening instrument widely used in epidemiological research to assess behavioral and emotional problems in children aged 4 to 17 years. Each item is scored on a three-point scale (0 = not true, 1 = somewhat true, 2 = certainly true), with selected items reverse-scored so that higher values consistently indicate greater difficulty. The 25 items yield five subscales of five items each: emotional symptoms, peer relationship problems, conduct problems, hyperactivity/inattention, and prosocial behavior. Each subscale ranges from 0 to 10 points.

The present study focused on the four difficulty subscales. Following established practice (Goodman et al., 2010), three composite indices were also calculated: a total difficulties score (sum of the four difficulty subscales, range 0 to 40), an internalizing problems score (emotional symptoms plus peer problems, range 0 to 20), and an externalizing problems score (conduct problems plus hyperactivity/inattention, range 0 to 20). The SDQ was completed by the primary caregiver at both Sweep 4 (age 7) and Sweep 6 (age 14). Sweep 4 SDQ scores served as baseline covariates, and Sweep 6 SDQ scores served as the outcome variables.

Peer problems at age 14 was designated as the primary outcome on the basis of prior MCS literature consistently identifying this subscale as the dimension most strongly associated with childhood MVPA (Ahn et al., 2018; Werneck et al., 2025). The remaining SDQ subscales and composite indices (emotional symptoms, conduct problems, hyperactivity/inattention, total difficulties, internalizing problems, and externalizing problems) were treated as secondary outcomes. Sex-stratified analyses were considered exploratory.

### 2.5 Covariates

Potential confounders were selected on the basis of prior literature (Ahn et al., 2018; Werneck et al., 2025) and directed acyclic graph reasoning (the DAG is presented in Supplementary Figure S1). The following variables, measured at Sweep 4, were included as covariates in all models: child age (in years), sex (male or female), ethnicity (White vs. non-White), household income (above or below 60% of the national median), highest parental educational qualification (coded according to the National Vocational Qualification framework as levels 0 through 5), and parental psychological distress, assessed using the six-item Kessler Psychological Distress Scale (K6; Kessler et al., 2002). The corresponding SDQ subscale score at age 7 was included as a covariate in each model to adjust for baseline mental health status.

### 2.6 Statistical analysis

#### 2.6.1 Compositional data analysis

Because the three activity components (SB, LPA, MVPA) are subject to a constant-sum constraint (they must sum to total daily wear time), they cannot be entered as independent predictors in a standard regression model without introducing multicollinearity and violating the assumptions of unconstrained sample space (Aitchison, 1982). To address this, the activity composition was transformed using the isometric log-ratio (ILR) method (Egozcue et al., 2003), which maps the three-part composition onto two orthogonal coordinates:


$$\begin{array}{l} z1=\text{sqrt}{\left(2/3\right)}^{*}ln\left[SB/sqrt\left(LPA^{*}MVPA\right)\right] \end{array}$$

$$\begin{array}{l} z2=\text{sqrt}{\left(1/2\right)}^{*}ln\left(LPA/MVPA\right) \end{array}$$

The first coordinate (*z*1) captures the relative proportion of SB to the geometric mean of the two active behaviors (LPA and MVPA). The second coordinate (*z*2) captures the relative proportion of LPA to MVPA. These two ILR coordinates were then entered as predictors in multiple linear regression models, with each SDQ outcome at age 14 as the dependent variable, adjusting for baseline SDQ at age 7 and all covariates listed above.

The activity composition was expressed in minutes per day and entered into the ILR transformation without prior closure to proportions. The ILR transformation is scale-invariant, producing identical coordinates whether the input is expressed in absolute minutes or proportions of total wear time (Aitchison, 1982). The total duration underlying each individual's composition was the mean daily accelerometer wear time, which varied across participants (mean = 735.1, SD = 58.5 min/day). Because the CoDA framework inherently accounts for the relative nature of the composition, differences in total wear time do not bias the ILR coordinates. No participants had zero values in any activity component (minimum MVPA = 3.01 min/day), and therefore no zero-replacement procedure was required.

Total accelerometer wear time was not included as a covariate in the CoDA models because the compositional approach operates on the relative distribution of time across activity categories, rendering such adjustment unnecessary (Dumuid et al., 2018). In the conventional regression sensitivity analysis (Section 2.6.4), total wear time was likewise not included because the simultaneous entry of SB and MVPA, together with the implicitly omitted LPA, already partitions total wear time.

#### 2.6.2 Compositional isotemporal substitution

To translate the regression results into a more interpretable metric, we applied the compositional isotemporal substitution model (Dumuid et al., 2019). This approach estimates the predicted change in an outcome variable when a fixed duration of time is reallocated from one activity component to another, holding the remaining component and total wear time constant. The geometric mean composition of the full sample (i.e., the component-wise geometric mean of SB, LPA, and MVPA across all 5,145 participants) served as the reference point for the overall analysis. For sex-stratified analyses, the same full-sample geometric mean was used as the reference to ensure comparability across subgroups. We modeled the reallocation of 15 min per day across three substitution scenarios: SB replaced by MVPA, LPA replaced by MVPA, and SB replaced by LPA. For each scenario, the difference in predicted SDQ scores between the reallocated and reference compositions was computed, along with 95% confidence intervals derived using the delta method. The 15-min increment was selected because it represents a realistic and policy-relevant behavioral change target that has been widely adopted in the literature (Ahn et al., 2018; Dumuid et al., 2018).

#### 2.6.3 Sex-stratified analysis

Given prior evidence that the associations between physical activity and mental health may differ between boys and girls (Ahn et al., 2018; Werneck et al., 2025), all CoDA regression and isotemporal substitution analyses were conducted separately for the full sample, for boys, and for girls.

#### 2.6.4 Sensitivity analysis

As a sensitivity analysis and a form of analytical triangulation (Herold et al., 2026), we fitted conventional multiple linear regression models in which raw MVPA and SB (expressed as minutes per day) were entered simultaneously as continuous predictors alongside the same set of covariates; LPA was the implicitly omitted behavior, meaning that the MVPA coefficient represents the expected change in the outcome when MVPA increases by one unit and LPA decreases by the same amount, holding SB constant. Total accelerometer wear time was not included as a covariate in these models because SB and MVPA together with LPA already partition the total wear time. This allowed us to assess whether the direction and significance of the observed associations were consistent across the two analytic approaches and to facilitate comparison with prior MCS-based studies that employed this modeling strategy.

#### 2.6.5 Effect modification by sex

To formally test whether the associations between activity composition and mental health outcomes differed between boys and girls, interaction terms between each ILR coordinate and sex (*z*1 × sex, *z*2 × sex) were added to the regression models. The statistical significance of the interaction terms was evaluated to determine whether sex-stratified analyses were warranted.

#### 2.6.6 Adjustment for multiple comparisons

Given the multiple SDQ outcomes, substitution scenarios, and sex-stratified subgroups examined, the Benjamini-Hochberg false discovery rate (FDR) procedure was applied to control for multiple comparisons. Both unadjusted and FDR-adjusted *p*-values are reported. Findings are interpreted primarily on the basis of the FDR-adjusted results.

#### 2.6.7 Dose-response analysis

To explore dose-response patterns, isotemporal substitution analyses were repeated using 5-min and 10-min reallocation intervals in addition to the primary 15-min interval.

All analyses were performed using Python 3.12 (Python Software Foundation (PSF), Wilmington, United States) with the statsmodels (version 0.14) and scipy libraries. Statistical significance was set at a two-sided alpha of 0.05.

## 3 Results

### 3.1 Sample characteristics

The final analytic sample comprised 5,145 children [2,475 boys [48.1%] and 2,670 girls [51.9%]]. The mean age at Sweep 4 was 7.21 years (SD = 0.24). The majority of the sample was of White ethnicity (90.4%), and 81.4% of families reported a household income above 60% of the national median. Descriptive statistics for physical activity variables indicated that children accumulated an average of 393.8 (SD = 65.0) minutes per day of sedentary time, 280.4 (SD = 40.9) minutes per day of LPA, and 61.9 (SD = 22.0) minutes per day of MVPA, with a mean total accelerometer wear time of 735.1 (SD = 58.5) minutes per day. The mean SDQ total difficulties score increased from 6.77 (SD = 5.31) at age 7 to 7.35 (SD = 5.64) at age 14. Full descriptive statistics for all study variables are presented in Table 1.

**Table 1:** Baseline characteristics of the analytic sample (*N* = 5,145).

Variable	Mean ±SD/*n* (%)
Age (years)	7.21 ± 0.24
Sedentary behavior, SB (minutes/day)	392.88 ± 65.23
LPA (minutes/day)	280.38 ± 40.86
MPA (minutes/day)	42.03 ± 13.09
VPA (minutes/day)	19.84 ± 10.65
MVPA (minutes/day)	61.87 ± 21.99
Total wear time (minutes/day)	735.13 ± 58.47
Emotional symptoms (age 7)	1.68 ± 1.91
Peer problems (age 7)	1.16 ± 1.55
Conduct problems (age 7)	1.16 ± 1.40
Hyperactivity/inattention (age 7)	2.78 ± 2.33
SDQ total difficulties (age 7)	6.77 ± 5.31
Emotional symptoms (age 14)	1.89 ± 2.11
Peer problems (age 14)	1.55 ± 1.74
Conduct problems (age 14)	1.21 ± 1.48
Hyperactivity/inattention (age 14)	2.70 ± 2.34
SDQ total difficulties (age 14)	7.35 ± 5.64
Sex (male), *n* (%)	2,475 (48.1%)
White ethnicity, *n* (%)	4,617 (89.7%)
Income above 60th percentile, *n* (%)	4,160 (80.9%)
Parental education NVQ Level 0	364 (7.1%)
Parental education NVQ Level 1	283 (5.5%)
Parental education NVQ Level 2	1,292 (25.1%)
Parental education NVQ Level 3	765 (14.9%)
Parental education NVQ Level 4	2,037 (39.6%)
Parental education NVQ Level 5	404 (7.9%)
Parental psychological distress (K6)	2.77 ± 3.42

SB, sedentary behavior; LPA, light-intensity physical activity; MPA, moderate-intensity physical activity; VPA, vigorous-intensity physical activity; MVPA, moderate-to-vigorous-intensity physical activity. MPA and VPA were combined into MVPA for all compositional data analyses. SDQ, Strengths and Difficulties Questionnaire; K6, Kessler Psychological Distress Scale.

A comparison of baseline physical activity characteristics between the analytic sample (*n* = 5,145) and the excluded accelerometer subsample (*n* = 1,530) revealed no significant differences in SB (392.9 vs. 396.3 min/day, *p* = 0.082) or LPA (280.4 vs. 281.9 min/day, *p* = 0.196). A small but statistically significant difference was observed for MVPA (61.9 vs. 63.7 min/day, *p* = 0.006), though the magnitude of this difference (1.8 min/day) is unlikely to be practically meaningful.

### 3.2 Compositional regression analysis

Table 2 presents the associations between the ILR coordinates and SDQ outcomes at age 14, adjusted for baseline SDQ scores at age 7 and all covariates. After FDR correction, the second ILR coordinate (*z*2), representing the log-ratio of LPA relative to MVPA, remained positively associated with peer problems [beta = 0.273, 95% CI [0.061, 0.485], p_FDR = 0.040], indicating that a higher proportion of LPA relative to MVPA at age 7 predicted greater peer difficulties at age 14.

**Table 2:** Compositional regression: ILR coordinate associations with SDQ outcomes at age 14 (*N* = 5,145).

Outcome	ILR coordinate	β	SE	95% CI	*p*	p _FDR
Emotional symptoms	SB vs. active	−0.073	0.110	[−0.289, 0.143]	0.509	0.549
Emotional symptoms	LPA vs. MVPA	0.128	0.127	[−0.121, 0.376]	0.314	0.466
Peer problems	SB vs. active	−0.079	0.094	[−0.263, 0.105]	0.399	0.466
Peer problems	LPA vs. MVPA	0.273	0.108	[0.061, 0.485]	0.012^*^	0.040
Conduct problems	SB vs. active	−0.180	0.077	[−0.330, −0.029]	0.020^*^	0.055
Conduct problems	LPA vs. MVPA	0.117	0.088	[−0.056, 0.290]	0.185	0.323
Hyperactivity/inattention	SB vs. active	−0.493	0.111	[−0.710, −0.276]	< 0.001^***^	< 0.001
Hyperactivity/inattention	LPA vs. MVPA	0.072	0.126	[−0.175, 0.319]	0.567	0.567
Internalizing problems	SB vs. active	−0.149	0.168	[−0.478, 0.179]	0.374	0.466
Internalizing problems	LPA vs. MVPA	0.359	0.193	[−0.019, 0.737]	0.063	0.146
Externalizing problems	SB vs. active	−0.573	0.155	[−0.877, −0.269]	< 0.001^***^	0.002
Externalizing problems	LPA vs. MVPA	0.157	0.177	[−0.189, 0.503]	0.374	0.466
SDQ total difficulties	SB vs. active	−0.651	0.257	[−1.156, −0.147]	0.011^*^	0.040
SDQ total difficulties	LPA vs. MVPA	0.458	0.295	[−0.121, 1.036]	0.121	0.242

*z*1, ILR coordinate of SB relative to the geometric mean of LPA and MVPA; *z*_2_, ILR coordinate of LPA relative to MVPA. All models adjusted for baseline SDQ at age 7, age, sex, ethnicity, family income, parental education, and K6. ^*^p < 0.05, ^***^p < 0.001 for unadjusted *p*-values; p_FDR values are Benjamini-Hochberg FDR-adjusted.

The first ILR coordinate (*z*1), representing the log-ratio of SB relative to the geometric mean of LPA and MVPA, remained negatively associated after FDR correction with hyperactivity/inattention [beta = −0.493, 95% CI [−0.710, −0.276], p_FDR < 0.001], externalizing problems [beta = −0.573, 95% CI [−0.877, −0.269], p_FDR = 0.002], and total difficulties [beta = −0.651, 95% CI [−1.156, −0.147], p_FDR = 0.040]. Thus, a higher proportion of SB relative to active time at age 7 was associated with lower externalizing scores. Neither ILR coordinate was significantly associated with emotional problems or internalizing problems after FDR correction.

### 3.3 Isotemporal substitution analysis

Table 3 and Figure 1 present the modeled estimates of hypothetical reallocations of 15 min per day between activity types on each SDQ outcome at age 14.

**Table 3:** Compositional isotemporal substitution: estimated change in SDQ outcomes associated with 15-min daily reallocation (*N* = 5,145).

Outcome	Substitution	Δ	SE	95% CI	*p*	p _FDR
Emotional symptoms	SB → MVPA	−0.012	0.016	[−0.044, 0.021]	0.480	0.560
Emotional symptoms	LPA → MVPA	−0.021	0.022	[−0.063, 0.022]	0.343	0.479
Emotional symptoms	SB → LPA	0.009	0.010	[−0.010, 0.027]	0.365	0.479
Peer problems	SB → MVPA	−0.034	0.014	[−0.062, −0.007]	0.014^*^	0.059
Peer problems	LPA → MVPA	−0.050	0.019	[−0.086, −0.013]	0.008^**^	0.040
Peer problems	SB → LPA	0.014	0.008	[−0.002, 0.030]	0.077	0.161
Conduct problems	SB → MVPA	0.004	0.011	[−0.019, 0.026]	0.754	0.802
Conduct problems	LPA → MVPA	−0.011	0.015	[−0.041, 0.019]	0.472	0.560
Conduct problems	SB → LPA	0.014	0.007	[0.001, 0.027]	0.036^*^	0.107
Hyperactivity/inattention	SB → MVPA	0.050	0.016	[0.018, 0.082]	0.002^**^	0.024
Hyperactivity/inattention	LPA → MVPA	0.021	0.022	[−0.022, 0.063]	0.342	0.479
Hyperactivity/inattention	SB → LPA	0.029	0.010	[0.010, 0.048]	0.002^**^	0.024
Internalizing problems	SB → MVPA	−0.040	0.025	[−0.089, 0.009]	0.113	0.216
Internalizing problems	LPA → MVPA	−0.062	0.033	[−0.127, 0.003]	0.061	0.143
Internalizing problems	SB → LPA	0.021	0.015	[−0.007, 0.050]	0.141	0.247
Externalizing problems	SB → MVPA	0.047	0.023	[0.002, 0.092]	0.041^*^	0.108
Externalizing problems	LPA → MVPA	0.009	0.030	[−0.050, 0.068]	0.764	0.802
Externalizing problems	SB → LPA	0.037	0.013	[0.010, 0.063]	0.006^**^	0.040
SDQ total difficulties	SB → MVPA	0.008	0.038	[−0.067, 0.082]	0.840	0.840
SDQ total difficulties	LPA → MVPA	−0.046	0.051	[−0.145, 0.053]	0.362	0.479
SDQ total difficulties	SB → LPA	0.052	0.022	[0.008, 0.096]	0.020^*^	0.069

Positive values indicate worsening; negative values indicate improvement. Reference composition: component-wise geometric mean (SB = 387.7, LPA = 277.4, MVPA = 58.0 min/day). All models adjusted for baseline SDQ at age 7, age, sex, ethnicity, family income, parental education, and K6. ^*^p < 0.05, ^**^p < 0.01, for unadjusted *p*-values; p_FDR values are Benjamini-Hochberg FDR-adjusted.

**Figure 1:**
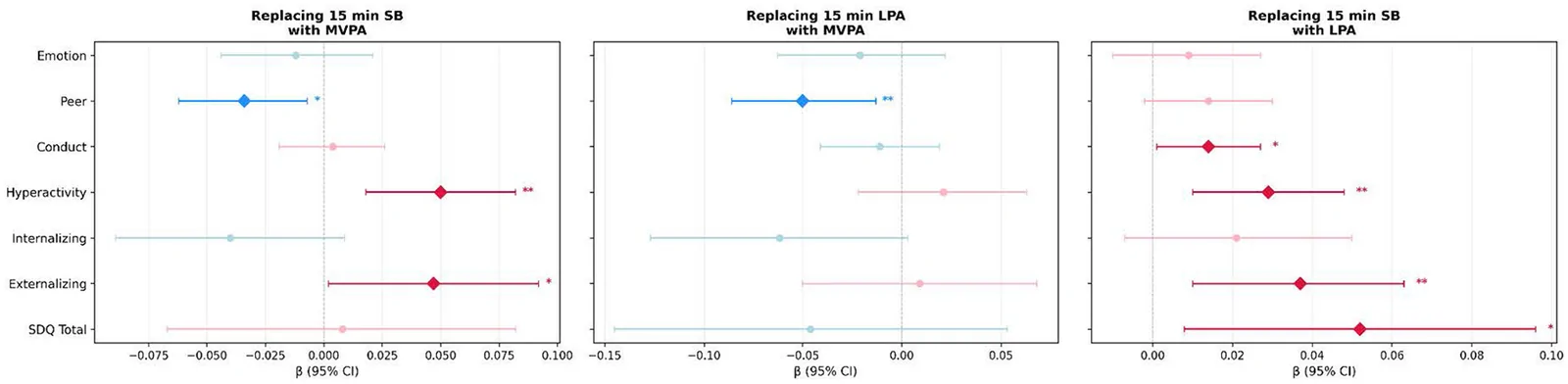
Isotemporal substitution effects on 14-year SDQ outcomes (15-min reallocation, adjusted for baseline SDQ and covariates, *N* = 5,145).

After FDR correction, reallocating 15 min from LPA to MVPA remained associated with fewer peer problems [delta = −0.050, 95% CI [−0.086, −0.013], p_FDR = 0.040]. The association for SB-to-MVPA reallocation was statistically significant before correction [delta = −0.034, 95% CI [−0.062, −0.007], *p* = 0.014] but did not survive FDR adjustment (p_FDR = 0.059). Neither substitution scenario was associated with internalizing problems after FDR correction.

For externalizing-related outcomes, SB-to-MVPA reallocation remained associated with higher hyperactivity/inattention scores after FDR correction [delta = 0.050, 95% CI [0.018, 0.082], p_FDR = 0.024], whereas its association with externalizing problems did not survive correction (p_FDR = 0.108). SB-to-LPA reallocation remained associated with higher hyperactivity/inattention [delta = 0.029, 95% CI [0.010, 0.048], p_FDR = 0.024] and externalizing problems [delta = 0.037, 95% CI [0.010, 0.063], p_FDR = 0.040]. LPA-to-MVPA reallocation was not associated with hyperactivity.

No significant associations were observed for emotional problems under any substitution scenario.

### 3.4 Sex-stratified isotemporal substitution analysis

Sex-stratified results are presented in Table 4 and Figure 2. Pronounced differences emerged between boys and girls. Among boys (*n* = 2,475), reallocating 15 minutes from LPA to MVPA was associated with significant reductions in peer problems (delta = −0.084, 95% CI [−0.122, −0.045], *p* < 0.001) and internalizing problems (delta = −0.087, 95% CI [−0.152, −0.023], *p* = 0.008). The effect sizes for boys were approximately 1.5 to 1.7 times larger than those observed in the full sample. Reallocating 15 minutes from SB to MVPA was similarly associated with reduced peer problems in boys (delta = −0.055, 95% CI [−0.089, −0.021], *p*= 0.002). At the same time, increases in hyperactivity (SB to MVPA: delta = 0.092, *p* < 0.001; LPA to MVPA: delta = 0.064, *p*= 0.007) and externalizing problems (SB to MVPA: delta = 0.085, *p*= 0.004) were also observed among boys.

**Table 4:** Sex-stratified isotemporal substitution: effects of SB → MVPA and LPA → MVPA (15 min/day).

Sex	Outcome	Substitution	Δ	95% CI	*p*
Boys	Emotional symptoms	SB → MVPA	−0.005	[−0.046, 0.037]	0.825
Boys	Emotional symptoms	LPA → MVPA	−0.021	[−0.073, 0.031]	0.452
Boys	Peer problems	SB → MVPA	−0.063	[−0.103, −0.023]	0.002^**^
Boys	Peer problems	LPA → MVPA	−0.097	[−0.149, −0.045]	< 0.001^***^
Boys	Conduct problems	SB → MVPA	−0.001	[−0.035, 0.034]	0.975
Boys	Conduct problems	LPA → MVPA	−0.014	[−0.055, 0.028]	0.539
Boys	Hyperactivity/inattention	SB → MVPA	0.081	[0.032, 0.129]	0.001^**^
Boys	Hyperactivity/inattention	LPA → MVPA	0.045	[−0.018, 0.108]	0.162
Boys	Internalizing problems	SB → MVPA	−0.054	[−0.120, 0.012]	0.113
Boys	Internalizing problems	LPA → MVPA	−0.103	[−0.190, −0.016]	0.021^*^
Boys	Externalizing problems	SB → MVPA	0.074	[0.006, 0.142]	0.033^*^
Boys	Externalizing problems	LPA → MVPA	0.031	[−0.056, 0.118]	0.491
Boys	SDQ total difficulties	SB → MVPA	0.028	[−0.078, 0.133]	0.609
Boys	SDQ total difficulties	LPA → MVPA	−0.058	[−0.186, 0.071]	0.419
Girls	Emotional symptoms	SB → MVPA	−0.016	[−0.068, 0.037]	0.527
Girls	Emotional symptoms	LPA → MVPA	−0.017	[−0.082, 0.048]	0.612
Girls	Peer problems	SB → MVPA	−0.001	[−0.042, 0.041]	0.981
Girls	Peer problems	LPA → MVPA	0.004	[−0.048, 0.056]	0.876
Girls	Conduct problems	SB → MVPA	0.007	[−0.027, 0.040]	0.661
Girls	Conduct problems	LPA → MVPA	−0.009	[−0.052, 0.033]	0.653
Girls	Hyperactivity/inattention	SB → MVPA	0.027	[−0.015, 0.069]	0.217
Girls	Hyperactivity/inattention	LPA → MVPA	0.001	[−0.053, 0.054]	0.987
Girls	Internalizing problems	SB → MVPA	−0.017	[−0.089, 0.055]	0.638
Girls	Internalizing problems	LPA → MVPA	−0.012	[−0.107, 0.082]	0.799
Girls	Externalizing problems	SB → MVPA	0.025	[−0.033, 0.083]	0.408
Girls	Externalizing problems	LPA → MVPA	−0.010	[−0.085, 0.066]	0.812
Girls	SDQ total difficulties	SB → MVPA	0.003	[−0.098, 0.105]	0.951
Girls	SDQ total difficulties	LPA → MVPA	−0.021	[−0.164, 0.122]	0.771

Boys *n* = 2,475; Girls *n* = 2,670. Reference: full-sample geometric mean (SB = 387.7, LPA = 277.4, MVPA = 58.0 min/day). All models adjusted for baseline SDQ at age 7, age, ethnicity, family income, parental education, and K6. ^*^p < 0.05, ^**^p < 0.01, ^***^p < 0.001.

**Figure 2:**
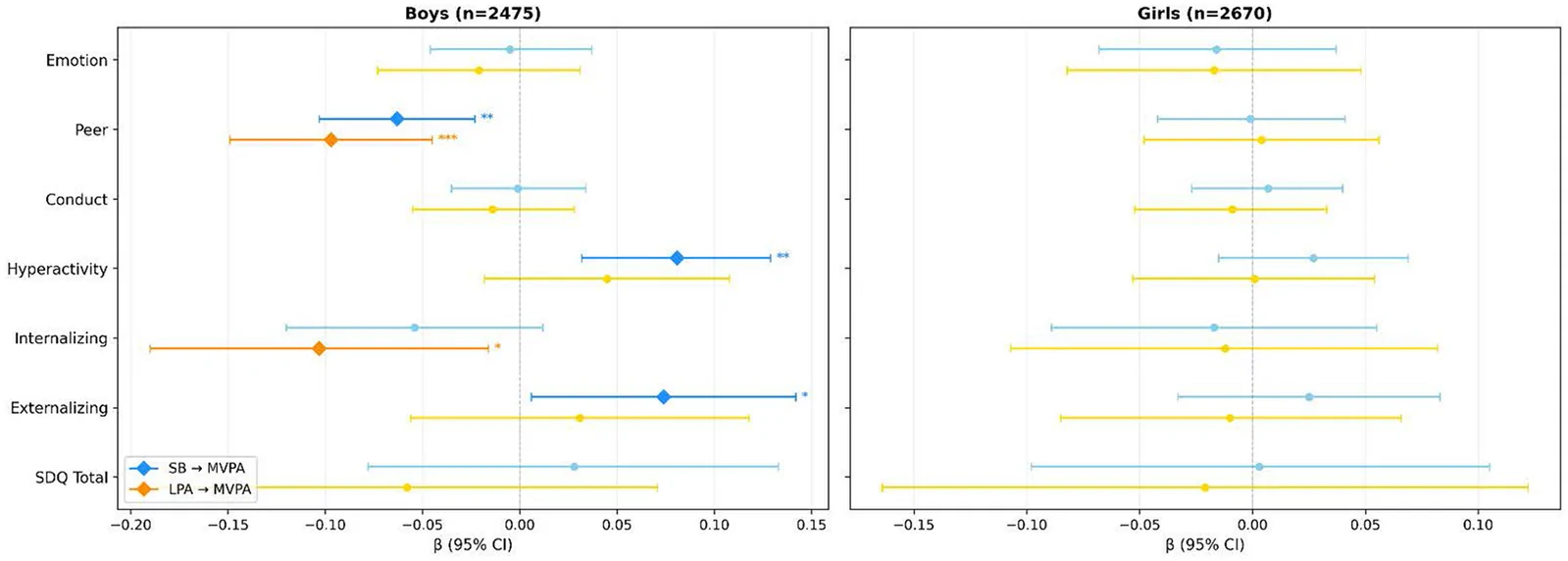
Sex-stratified isotemporal substitution effects (15-min reallocation) adjusted for baseline SDQ, age, ethnicity, income, parental education, parental distress.

Among girls (*n* = 2,670), none of the substitution scenarios yielded statistically significant associations with any SDQ outcome. The point estimates for peer problems and internalizing problems were close to zero (e.g., LPA to MVPA on peer problems: delta = 0.006, *p* = 0.801; on internalizing problems: delta = 0.001, *p* = 0.974), indicating that the protective associations identified in the full sample were driven predominantly by the male subsample.

### 3.5 Formal sex interaction tests

Formal interaction tests between sex and the ILR coordinates revealed one statistically significant interaction: the *z*2 (LPA vs. MVPA) × sex interaction was significant for peer problems [β = 0.624, 95% CI [0.202, 1.047], *p* = 0.004], indicating that the association between the LPA-to-MVPA ratio and peer problems differed significantly between boys and girls. The z2 × sex interaction for internalizing problems approached but did not reach conventional significance [β = 0.699, 95% CI [−0.055, 1.452], *p* = 0.069]. No other interaction terms were statistically significant (all *p* > 0.09; see Supplementary Table S1). These results provide formal statistical support for sex differences in the MVPA-peer problems association but do not confirm sex-dependent effects for other SDQ outcomes.

### 3.6 Multiple comparison adjustment

After Benjamini-Hochberg FDR correction of the CoDA regression results, the associations of z1 with hyperactivity/inattention (p_FDR < 0.001), externalizing problems (p_FDR = 0.002), and total difficulties (p_FDR = 0.040), and the association of z2 with peer problems (p_FDR = 0.040) remained statistically significant. The association of z1 with conduct problems (unadjusted *p* = 0.020) did not survive FDR correction (p_FDR = 0.055). For the 15-min isotemporal substitution results, FDR correction preserved the significance of LPA → MVPA on peer problems (p_FDR = 0.040), SB → MVPA on hyperactivity (p_FDR = 0.024), SB → LPA on hyperactivity (p_FDR = 0.024), and SB → LPA on externalizing problems (p_FDR = 0.040). However, SB → MVPA on peer problems (unadjusted p = 0.014; p_FDR = 0.059) and several other previously significant associations did not survive correction (see Tables 2, 3).

### 3.7 Dose-response patterns across reallocation intervals

Isotemporal substitution analyses using 5-min and 10-min intervals yielded results that were directionally consistent with the 15-min analyses, with effect sizes scaling approximately proportionally to the reallocation magnitude. For example, the estimated reduction in peer problems associated with LPA → MVPA reallocation was −0.017 (5 min), −0.034 (10 min), and −0.050 (15 min), demonstrating a consistent dose-response gradient. Full results across all intervals are presented in Supplementary Table S2.

### 3.8 Descriptive dose-response patterns

To provide a complementary perspective on the activity-mental health gradient, unadjusted mean SDQ scores at age 14 were plotted across quartiles of MVPA at age 7 (Figure 4). Emotional problems, peer problems, and internalizing problems exhibited a decreasing trend from the lowest to the highest MVPA quartile, consistent with the direction of the isotemporal substitution results. Hyperactivity scores displayed the opposite pattern, increasing across MVPA quartiles. These descriptive patterns should be interpreted with caution, as they are unadjusted and do not account for confounding.

**Figure 3:**
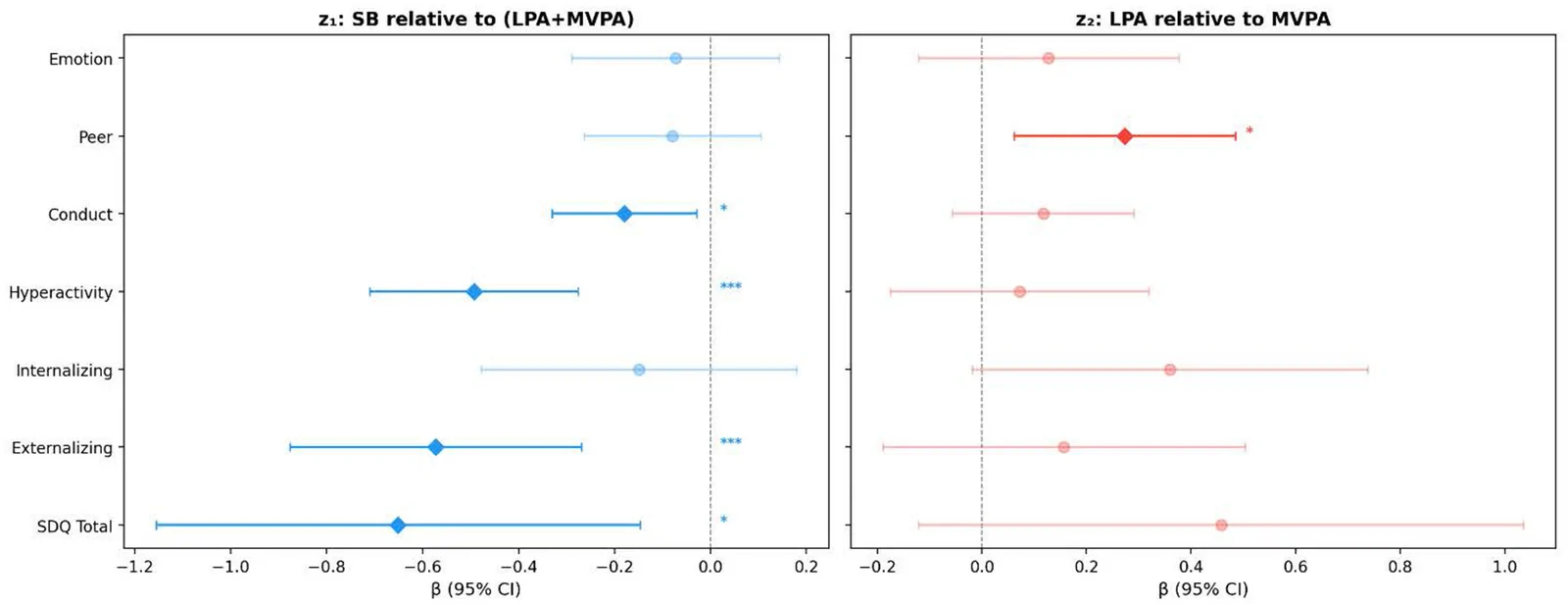
Compositional dats analysis: ILR coordinate associations with 14-year old SDQ (*N* = 5,145) adjusted for baseline SDQ and covariates.

**Figure 4:**
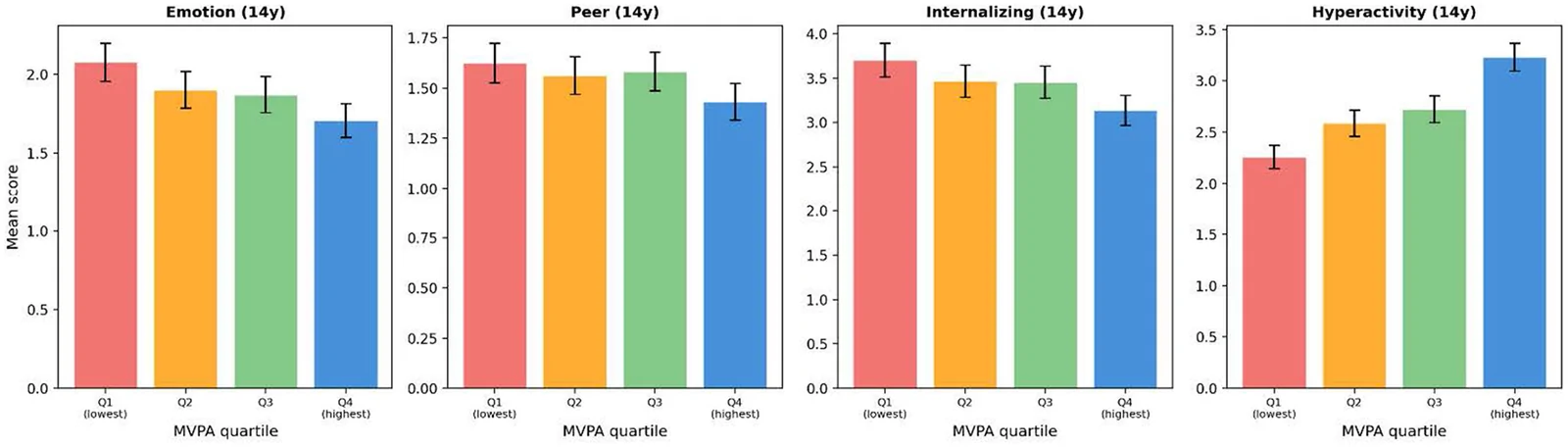
SDQ outcomes at age 14 by MVPA quartile at age 7 (unadjusted means).

### 3.9 Sensitivity analysis

Table 5 reports the results of the conventional multiple linear regression models. Each 15-min increment in daily MVPA was associated with a significant reduction in peer problems [beta = −0.034, 95% CI [−0.066, −0.003], *p* = 0.034] and significant increases in hyperactivity [beta = 0.052, 95% CI [0.016, 0.089], *p* = 0.005]. Each 15-min increment in daily SB was associated with reduced hyperactivity [beta = −0.017, 95% CI [−0.029, −0.005], *p* = 0.005], conduct problems [beta = −0.013, 95% CI [−0.022, −0.005], *p* = 0.002], externalizing problems [beta = −0.027, 95% CI [−0.044, −0.010], *p* = 0.002], and total difficulties [beta = −0.036, 95% CI [−0.064, −0.008], *p* = 0.013].

**Table 5:** Conventional linear regression: associations with SDQ outcomes at age 14 (per 15-min daily increment; *N* = 5,145).

Outcome	Predictor	β	SE	95% CI	*p*
Emotional symptoms	MVPA	−0.024	0.019	[−0.061, 0.013]	0.202
Emotional symptoms	SB	−0.009	0.006	[−0.021, 0.003]	0.125
Peer problems	MVPA	−0.034	0.016	[−0.066, −0.003]	0.034^*^
Peer problems	SB	−0.004	0.005	[−0.014, 0.006]	0.440
Conduct problems	MVPA	−0.012	0.013	[−0.038, 0.014]	0.367
Conduct problems	SB	−0.013	0.004	[−0.022, −0.005]	0.002^**^
Hyperactivity/inattention	MVPA	0.052	0.019	[0.016, 0.089]	0.005^**^
Hyperactivity/inattention	SB	−0.017	0.006	[−0.029, −0.005]	0.005^**^
SDQ total difficulties	MVPA	−0.010	0.044	[−0.096, 0.075]	0.813
SDQ total difficulties	SB	−0.036	0.014	[−0.064, −0.008]	0.013^*^
Internalizing problems	MVPA	−0.048	0.029	[−0.104, 0.008]	0.093
Internalizing problems	SB	−0.012	0.009	[−0.030, 0.006]	0.203
Externalizing problems	MVPA	0.034	0.026	[−0.018, 0.085]	0.197
Externalizing problems	SB	−0.027	0.009	[−0.044, −0.010]	0.002^**^

MVPA and SB entered simultaneously; LPA implicitly omitted. The MVPA coefficient = expected change when MVPA +15 min and LPA −15 min, holding SB constant. All models adjusted for baseline SDQ at age 7, age, sex, ethnicity, family income, parental education, and K6. ^*^p < 0.05, ^**^p < 0.01.

The direction and statistical significance of these associations were consistent with those obtained from the CoDA and isotemporal substitution analyses. MVPA was associated with lower peer problems and higher externalizing and hyperactivity scores under both analytic frameworks, and higher SB was associated with lower externalizing outcomes in both sets of models. This convergence across methods supports the robustness of the principal findings and indicates that the observed associations are not an artifact of a particular analytic approach.

## 4 Discussion

Using longitudinal data from the UK Millennium Cohort Study and a compositional data analysis framework, this study examined prospective associations between the composition of objectively measured physical activity at age 7 and multiple dimensions of mental health at age 14. Two principal patterns of association emerged. First, after FDR correction, reallocating time from LPA to MVPA was associated with fewer peer problems. Second, reallocations from SB to MVPA and from SB to LPA were associated with higher hyperactivity scores, and SB-to-LPA reallocation was also associated with higher externalizing scores. Thus, the direction of association varied across mental health dimensions. Sex-stratified analyses revealed that the associations with peer and internalizing problems appeared stronger among boys than among girls. Conventional regression models yielded results that were directionally consistent with those from the CoDA framework, supporting the robustness of these findings.

### 4.1 Protective associations of MVPA with peer and internalizing problems

The finding that a greater relative share of MVPA in the daily activity composition was associated with fewer peer problems at age 14 is broadly consistent with prior MCS-based studies. Ahn et al. (2018) reported that each additional 15 minutes per day of MVPA at age 7 predicted a reduction of 0.077 points in peer problems at age 11. The effect sizes observed in the present study were smaller in magnitude (0.036 to 0.049 points per 15-minutes reallocation), a difference that may reflect the longer follow-up interval used here. Extending the outcome measurement from age 11 to age 14 introduces a period of rapid social reorganization associated with the transition to secondary school and the onset of puberty, during which the influence of childhood activity patterns on peer functioning may attenuate (Sellers et al., 2019; Rubin et al., 2015). Werneck et al. (2025), who tracked MCS participants through ages 11, 14, and 17, also observed that MVPA at age 7 predicted lower peer problems, lending further support to the persistence of this association into adolescence. Wang and Wang (2026) reported a similar protective trend for peer difficulties in the full MCS sample, although their analysis did not extend to conduct or hyperactivity outcomes and therefore could not detect the divergent associations identified in the present study. Li et al. (2025b) observed that the association between MVPA and internalizing problems strengthened at the age 14 follow-up relative to age 11, with the peer problem component driving much of this effect. However, their sample was restricted to overweight and obese participants (*n* = 858), limiting generalizability to the broader pediatric population.

Several mechanisms operating at multiple levels may underlie the association between MVPA and reduced peer difficulties. At the psychosocial level, MVPA is more likely to occur in structured, interactive settings such as team sports and playground games, which provide children with repeated opportunities for cooperation, negotiation, and conflict resolution (Eime et al., 2013). Sustained participation in such activities may foster a sense of competence and social belonging that facilitates peer acceptance (Bailey, 2006). At the neurobiological level, Lubans et al. (2016) proposed that physical activity may promote neuroplasticity through multiple pathways, including upregulation of brain-derived neurotrophic factor (BDNF) and enhanced hippocampal function, processes that may support emotional regulation and reduce vulnerability to internalizing symptoms. However, it should be noted that these mechanisms are hypothetical in the present context, as the MCS accelerometer data cannot distinguish between physical activity types (e.g., aerobic vs. resistance activities), and the observed associations may operate through any combination of psychosocial, neurobiological, and behavioral pathways. The FDR-adjusted association of LPA-to-MVPA reallocation with fewer peer problems, together with the absence of a corresponding association for SB-to-LPA reallocation, is consistent with the hypothesis that physical activity intensity, rather than simply reducing sedentary time, may be a relevant parameter; this interpretation remains tentative.

### 4.2 Divergent associations with hyperactivity and externalizing problems

An important finding was that reallocating time to MVPA was concurrently associated with higher hyperactivity and externalizing scores. The CoDA regression confirmed this pattern: the *z*1 coordinate, reflecting the relative proportion of SB to active behaviors, was negatively associated with hyperactivity, conduct problems, and externalizing problems, meaning that children who spent proportionally more time sedentary at age 7 exhibited fewer externalizing difficulties at age 14. These results converge with Ahn et al. (2018), who reported that higher sedentary time was associated with lower hyperactivity scores, and with Werneck et al. (2025), who found that MVPA at age 7 predicted higher hyperactivity across adolescence among boys. Li et al. (2025) also observed a positive association between LPA and externalizing problems at age 11 in their overweight subsample. More recently, Cai et al. (2026) reported comparable patterns using a physical activity-sedentary time balance approach applied to MCS data. It is important to note that this pattern of associations between MVPA and externalizing outcomes has been observed primarily within the MCS dataset, and replication in other cohorts is needed before more robust conclusions can be drawn.

Interpreting these associations requires careful consideration of potential reverse causality and measurement characteristics. Children with elevated hyperactive or impulsive behavioral traits are likely to generate higher accelerometer counts as a direct consequence of their behavioral profile, producing an association between measured MVPA and subsequent hyperactivity that reflects behavioral continuity rather than a causal effect of physical activity on externalizing problems. This explanation is supported by the high temporal stability of hyperactivity symptoms across childhood and adolescence (Lahey et al., 2004) and by the observation that the association was strongest for the hyperactivity subscale, where the behavioral overlap with accelerometer-detected movement is most pronounced. The SDQ hyperactivity subscale includes items describing restlessness and difficulty remaining seated, behaviors that accelerometers cannot distinguish from volitional physical activity. This content overlap between the exposure and outcome measures may inflate the observed association. It is therefore important to apply a consistent causal standard to both directions of association. Although the associations with peer and internalizing problems may plausibly reflect benefits of MVPA, the same observational framework cannot rule out confounding or reverse causality for these outcomes either. The positive associations with hyperactivity and externalizing problems likely reflect, at least in part, confounding by pre-existing behavioral dispositions. Both favorable and unfavorable associations should be interpreted as model-predicted patterns rather than established causal effects.

### 4.3 Sex differences

Sex-stratified analyses suggested that the associations of MVPA with peer and internalizing problems were more pronounced among boys than among girls. In boys, the effect size for the LPA-to-MVPA reallocation on peer problems was approximately 1.9 times larger than that observed in the full sample, whereas in girls, no substitution scenario yielded a statistically significant association with any SDQ outcome. This finding is consistent with Ahn et al. (2018), who reported stronger MVPA-peer problem associations among boys, and with Werneck et al. (2025), whose sex-stratified models showed significant MVPA-peer problem links for boys but not girls. Systematic reviews have acknowledged that sex-specific evidence in this domain remains limited and inconclusive (Biddle et al., 2019).

Several factors may contribute to this pattern. First, boys at age 7 in the present sample accumulated more daily MVPA than girls (approximately 67 vs. 53 min per day in the MCS accelerometer data; Griffiths et al., 2013), providing a wider range of activity variation within which substitution effects can be detected. This greater variability may afford more statistical power to identify associations among boys, whereas the more compressed MVPA distribution among girls could attenuate detectable effects. Second, the types of MVPA engaged in by boys and girls may differ qualitatively. Boys are more likely to participate in team-based activities such as football and playground chasing games, which involve substantial peer interaction and social negotiation (Eime et al., 2013). Girls' MVPA may be distributed more evenly across individual and less socially embedded activities, potentially reducing the social mediation pathway linking physical activity intensity to peer outcomes. However, it must be emphasized that accelerometry measures movement intensity and duration rather than activity context or type, and these interpretations involving specific social mechanisms remain hypothetical. Third, it is possible that boys rely more heavily on physical play as a vehicle for emotional regulation and social bonding during middle childhood, such that changes in MVPA have a more direct bearing on their psychosocial adjustment. These explanations are not mutually exclusive, and the present data cannot adjudicate among them. Future research that incorporates contextual information about the type and social setting of physical activity will be needed to clarify the mechanisms underlying these sex differences.

### 4.4 Methodological contribution and sensitivity analysis

A notable strength of this study is its application of compositional data analysis and isotemporal substitution modeling to MCS longitudinal data. While CoDA has been increasingly applied in movement behavior research (Bourke et al., 2025; Kuzik et al., 2025), the specific contribution of the present analysis lies in the prospective examination of objectively measured childhood waking activity composition in relation to multidimensional psychological outcomes over a 7-year follow-up within a compositional reallocation framework. The four prior MCS-based studies examining physical activity and SDQ outcomes (Ahn et al., 2018; Li et al., 2025; Wang and Wang, 2026; Werneck et al., 2025) all relied on conventional regression approaches that do not account for the constant-sum constraint of daily time use. CoDA addresses this constraint through log-ratio transformation, and the isotemporal substitution extension translates model estimates into a metric that corresponds directly to a behavioral change scenario, thereby increasing the relevance of the findings for public health intervention design.

The convergence of the conventional regression sensitivity analysis with the CoDA results provides additional reassurance. Both approaches identified the same direction of association for all significant findings: MVPA was linked with lower peer problems and higher hyperactivity, while higher SB was associated with lower externalizing outcomes. This consistency indicates that the principal findings are not contingent on the choice of analytic framework. At the same time, the conventional models cannot specify the substitution reference (whether a reduction in SB, LPA, or both offsets the increase in MVPA), a limitation that underscores the added interpretive value of the compositional approach. Other design features that support the validity of these findings include the 7-year prospective follow-up, the use of accelerometry rather than self-report to assess physical activity, the adjustment for baseline SDQ scores and a broad set of sociodemographic confounders, and the large sample drawn from a nationally designed birth cohort.

### 4.5 Limitations

Several limitations should be acknowledged. First, despite the prospective design and adjustment for baseline mental health, the isotemporal substitution estimates represent observational associations derived from a mathematical reallocation model, not effects of an actual behavioral intervention. Establishing causality will require experimental or quasi-experimental designs. Second, physical activity was assessed at a single time point (age 7), and changes in activity patterns over the 7-year follow-up period could not be captured. Although children's physical activity levels show moderate tracking stability during middle childhood (Telama et al., 2014), a single measurement may not fully represent habitual activity across this developmental window. Third, approximately 60% of the original MCS accelerometer subsample was excluded due to missing SDQ, covariate, or wear-time data. Attrition analyses in the MCS have indicated that non-participants tend to come from more socioeconomically disadvantaged backgrounds (Griffiths et al., 2013), which may limit generalizability and introduce selection bias. Fourth, waist-worn accelerometers quantify the volume and intensity of bodily movement but cannot differentiate between activity types. The inability to distinguish team sports from solitary activities is a consequential limitation, given that the social context of physical activity is a plausible mediator of the associations with peer outcomes. Fifth, the SDQ was completed by parents, whose perceptions of their child's behavior may differ systematically from teacher ratings or adolescent self-reports. Multi-informant designs in future studies could help to address this source of measurement variability. Sixth, the present analysis focused on waking activity behaviors (SB, LPA, MVPA) without incorporating sleep into the compositional framework. Given the growing recognition of the 24-h movement behavior paradigm (Chastin et al., 2015; Dumuid et al., 2018), the exclusion of sleep represents a limitation, and future studies should consider the full 24-h activity composition. This broader perspective is relevant to attentional outcomes: among youth with moderate/severe ADHD, greater adherence to 24-h movement behavior guidelines was associated with fewer core symptoms and higher school engagement (Li et al., 2025a). Seventh, the classification of physical activity into intensity zones based on accelerometer count thresholds involves inherent assumptions, and the exact demarcation of intensity categories remains a matter of ongoing debate (Bishop et al., 2025). Furthermore, this study focused on intensity as a single parameter of the physical activity dose, whereas other parameters such as frequency, duration, and density, as well as their interactions with moderators such as sex, also warrant investigation (Herold et al., 2025a,b; Kobeissi et al., 2026). Eighth, accelerometer-derived SB encompasses both mentally passive and mentally active forms, which may have differential effects on mental health outcomes (Hallgren et al., 2020; Werneck et al., 2021a,b; Zou et al., 2024; Zhang et al., 2025; Mallawaarachchi et al., 2024). The inability to distinguish between these sedentary subtypes represents a further limitation. The temporal pattern of sitting may also matter, as interrupting prolonged sitting with exercise breaks can acutely improve cognitive function (Li et al., 2022). Future studies could combine device-based measures with functional near-infrared spectroscopy to examine neurophysiological pathways linking sedentary behavior to brain health (Li et al., 2026). Ninth, the MCS sampling design involves clustering, stratification, and differential non-response, and the present analyses did not incorporate survey weights or account for the complex sampling structure. Future studies using multiple imputation or inverse probability weighting would help to address this concern. Finally, although Benjamini-Hochberg FDR correction was applied to the primary analyses, some borderline findings (e.g., the association between SB-to-MVPA reallocation and peer problems) did not survive adjustment. Findings with *p*-values close to conventional thresholds should therefore be interpreted with particular caution.

### 4.6 Public health implications

From a public health perspective, the modeled effect sizes are modest at the individual level. A reallocation of 15 min per day from LPA to MVPA was associated with a reduction of approximately 0.05 points on the peer problems subscale. Given that this subscale ranges from 0 to 10 with a population standard deviation of approximately 1.7, the corresponding standardized effect size (Cohen's d) is approximately 0.03. While this magnitude may appear trivial for an individual child, population-level reasoning suggests otherwise. Rose (1985) argued that a small shift in the distribution of a risk factor across an entire population can yield a larger aggregate health benefit than a large shift confined to a high-risk subgroup. Applied to the present context, a nationwide policy that successfully increased daily MVPA by 15 min among school-aged children could potentially reduce the overall prevalence of peer difficulties at the population level. However, the current observational study cannot establish that such model-predicted differences would translate into meaningful population-level mental health improvements. The findings also carry implications for intervention design. Existing systematic reviews suggest that physical activity interventions can improve mental health in children (Hale et al., 2023; Andermo et al., 2020; Biddle et al., 2019), providing broader support for the promotion of MVPA during middle childhood. The suggestive sex differences observed here, if confirmed by replication in independent cohorts, may have implications for tailoring physical activity promotion strategies. For boys, encouraging participation in socially interactive MVPA may be a promising strategy. For girls, increasing physical activity intensity alone may be insufficient, and interventions may need to attend more deliberately to the social and motivational dimensions of physical activity participation.

## 5 Conclusion

Using a compositional data analysis framework applied to longitudinal data from 5,145 participants in the UK Millennium Cohort Study, this study found that modeled reallocation of daily time from LPA to MVPA at age 7 was prospectively associated with fewer peer problems at age 14. Reallocation from SB to MVPA and from SB to LPA was concurrently associated with higher hyperactivity scores, and SB-to-LPA reallocation was also associated with higher externalizing scores. Sex-stratified analyses suggested that the associations with peer and internalizing problems were more pronounced among boys, with formal interaction testing confirming sex differences for the MVPA-peer problems association (*p* = 0.004), though not for other outcomes. These findings indicate that the relationship between childhood physical activity composition and adolescent mental health is dimension-specific. The divergent pattern across mental health domains calls for careful interpretation, particularly regarding externalizing outcomes where reverse causality and measurement overlap are plausible explanations. From a research standpoint, the results highlight the potential relevance of physical activity intensity composition for youth mental health and underscore the need for experimental studies to establish whether increasing MVPA during middle childhood causally improves peer outcomes.

## Data Availability

The datasets presented in this study can be found in online repositories. The names of the repository/repositories and accession number(s) can be found below: https://cls.ucl.ac.uk/cls-studies/millennium-cohort-study/.
