# Supplementary material for: Reallocating childhood physical activity and sedentary time and adolescent mental health: a compositional data analysis of the UK Millennium Cohort Study
Source: Front Psychol. 2026 Sep 28;17:1932607. doi: 10.3389/fpsyg.2026.1932607 (PMC13654704; doi:10.3389/fpsyg.2026.1932607)
Supplement: Supplementary file 1 [file Supplementary_Tables.docx]

**Supplementary Table S1. Formal Sex × ILR Coordinate Interaction Tests (N = 5,145)**

| **Outcome** | **Interaction** | **β** | **SE** | **95% CI** | **p** |
| --- | --- | --- | --- | --- | --- |
| Emotional symptoms | z₁ × Sex | -0.209 | 0.219 | [-0.638, 0.221] | 0.341 |
| Emotional symptoms | z₂ × Sex | 0.118 | 0.253 | [-0.377, 0.614] | 0.640 |
| Peer problems | z₁ × Sex | -0.266 | 0.186 | [-0.631, 0.099] | 0.154 |
| Peer problems | z₂ × Sex | 0.624 | 0.215 | [0.202, 1.047] | 0.004** |
| Conduct problems | z₁ × Sex | 0.080 | 0.152 | [-0.219, 0.379] | 0.600 |
| Conduct problems | z₂ × Sex | -0.028 | 0.176 | [-0.373, 0.317] | 0.873 |
| Hyperactivity/inattention | z₁ × Sex | -0.365 | 0.217 | [-0.790, 0.061] | 0.093 |
| Hyperactivity/inattention | z₂ × Sex | -0.093 | 0.251 | [-0.585, 0.399] | 0.712 |
| Total difficulties | z₁ × Sex | -0.863 | 0.509 | [-1.861, 0.135] | 0.090 |
| Total difficulties | z₂ × Sex | 0.533 | 0.588 | [-0.619, 1.686] | 0.364 |
| Internalizing problems | z₁ × Sex | -0.532 | 0.333 | [-1.184, 0.121] | 0.110 |
| Internalizing problems | z₂ × Sex | 0.699 | 0.384 | [-0.055, 1.452] | 0.069 |
| Externalizing problems | z₁ × Sex | -0.285 | 0.304 | [-0.882, 0.312] | 0.350 |
| Externalizing problems | z₂ × Sex | -0.127 | 0.352 | [-0.817, 0.563] | 0.718 |

*Note: Sex coded as 1 = male, 0 = female. All models included main effects of z₁, z₂, and sex, plus covariates. *p < 0.05, **p < 0.01, ***p < 0.001.*

**Supplementary Table S2. Dose-Response: Isotemporal Substitution at 5, 10, and 15 Minutes (N = 5,145)**

| **Min** | **Outcome** | **Subst.** | **Δ** | **SE** | **95% CI** | **p** |
| --- | --- | --- | --- | --- | --- | --- |
| 5 | Emotional symptoms | SB→MVPA | -0.004 | 0.006 | [-0.016, 0.007] | 0.472 |
| 5 | Emotional symptoms | LPA→MVPA | -0.007 | 0.008 | [-0.022, 0.008] | 0.346 |
| 5 | Emotional symptoms | SB→LPA | 0.003 | 0.003 | [-0.003, 0.009] | 0.364 |
| 5 | Peer problems | SB→MVPA | -0.012 | 0.005 | [-0.022, -0.003] | 0.013* |
| 5 | Peer problems | LPA→MVPA | -0.017 | 0.007 | [-0.030, -0.005] | 0.007** |
| 5 | Peer problems | SB→LPA | 0.005 | 0.003 | [-0.001, 0.010] | 0.075 |
| 5 | Conduct problems | SB→MVPA | 0.001 | 0.004 | [-0.007, 0.009] | 0.786 |
| 5 | Conduct problems | LPA→MVPA | -0.004 | 0.005 | [-0.014, 0.007] | 0.495 |
| 5 | Conduct problems | SB→LPA | 0.005 | 0.002 | [0.000, 0.009] | 0.036* |
| 5 | Hyperactivity/inattention | SB→MVPA | 0.018 | 0.006 | [0.006, 0.029] | 0.003** |
| 5 | Hyperactivity/inattention | LPA→MVPA | 0.008 | 0.008 | [-0.007, 0.023] | 0.301 |
| 5 | Hyperactivity/inattention | SB→LPA | 0.010 | 0.003 | [0.003, 0.016] | 0.002** |
| 5 | Internalizing problems | SB→MVPA | -0.014 | 0.009 | [-0.032, 0.003] | 0.109 |
| 5 | Internalizing problems | LPA→MVPA | -0.022 | 0.012 | [-0.044, 0.001] | 0.062 |
| 5 | Internalizing problems | SB→LPA | 0.007 | 0.005 | [-0.002, 0.017] | 0.140 |
| 5 | Externalizing problems | SB→MVPA | 0.016 | 0.008 | [0.000, 0.032] | 0.048* |
| 5 | Externalizing problems | LPA→MVPA | 0.004 | 0.011 | [-0.017, 0.025] | 0.714 |
| 5 | Externalizing problems | SB→LPA | 0.012 | 0.005 | [0.003, 0.021] | 0.006** |
| 5 | SDQ total difficulties | SB→MVPA | 0.002 | 0.014 | [-0.025, 0.029] | 0.877 |
| 5 | SDQ total difficulties | LPA→MVPA | -0.016 | 0.018 | [-0.050, 0.019] | 0.382 |
| 5 | SDQ total difficulties | SB→LPA | 0.017 | 0.008 | [0.003, 0.032] | 0.020* |
| 10 | Emotional symptoms | SB→MVPA | -0.008 | 0.011 | [-0.030, 0.014] | 0.476 |
| 10 | Emotional symptoms | LPA→MVPA | -0.014 | 0.015 | [-0.043, 0.015] | 0.344 |
| 10 | Emotional symptoms | SB→LPA | 0.006 | 0.006 | [-0.007, 0.018] | 0.364 |
| 10 | Peer problems | SB→MVPA | -0.024 | 0.010 | [-0.043, -0.005] | 0.014* |
| 10 | Peer problems | LPA→MVPA | -0.034 | 0.013 | [-0.059, -0.009] | 0.008** |
| 10 | Peer problems | SB→LPA | 0.010 | 0.005 | [-0.001, 0.020] | 0.076 |
| 10 | Conduct problems | SB→MVPA | 0.002 | 0.008 | [-0.013, 0.018] | 0.770 |
| 10 | Conduct problems | LPA→MVPA | -0.007 | 0.010 | [-0.028, 0.013] | 0.483 |
| 10 | Conduct problems | SB→LPA | 0.010 | 0.005 | [0.001, 0.019] | 0.036* |
| 10 | Hyperactivity/inattention | SB→MVPA | 0.034 | 0.011 | [0.012, 0.057] | 0.002** |
| 10 | Hyperactivity/inattention | LPA→MVPA | 0.015 | 0.015 | [-0.014, 0.044] | 0.321 |
| 10 | Hyperactivity/inattention | SB→LPA | 0.020 | 0.006 | [0.007, 0.032] | 0.002** |
| 10 | Internalizing problems | SB→MVPA | -0.028 | 0.017 | [-0.061, 0.006] | 0.111 |
| 10 | Internalizing problems | LPA→MVPA | -0.042 | 0.023 | [-0.087, 0.002] | 0.061 |
| 10 | Internalizing problems | SB→LPA | 0.014 | 0.010 | [-0.005, 0.033] | 0.140 |
| 10 | Externalizing problems | SB→MVPA | 0.032 | 0.016 | [0.001, 0.063] | 0.044* |
| 10 | Externalizing problems | LPA→MVPA | 0.007 | 0.021 | [-0.034, 0.047] | 0.739 |
| 10 | Externalizing problems | SB→LPA | 0.024 | 0.009 | [0.007, 0.042] | 0.006** |
| 10 | SDQ total difficulties | SB→MVPA | 0.005 | 0.026 | [-0.047, 0.056] | 0.859 |
| 10 | SDQ total difficulties | LPA→MVPA | -0.031 | 0.035 | [-0.099, 0.037] | 0.372 |
| 10 | SDQ total difficulties | SB→LPA | 0.035 | 0.015 | [0.006, 0.064] | 0.020* |
| 15 | Emotional symptoms | SB→MVPA | -0.012 | 0.016 | [-0.044, 0.021] | 0.480 |
| 15 | Emotional symptoms | LPA→MVPA | -0.021 | 0.022 | [-0.063, 0.022] | 0.343 |
| 15 | Emotional symptoms | SB→LPA | 0.009 | 0.010 | [-0.010, 0.027] | 0.365 |
| 15 | Peer problems | SB→MVPA | -0.034 | 0.014 | [-0.062, -0.007] | 0.014* |
| 15 | Peer problems | LPA→MVPA | -0.050 | 0.019 | [-0.086, -0.013] | 0.008** |
| 15 | Peer problems | SB→LPA | 0.014 | 0.008 | [-0.002, 0.030] | 0.077 |
| 15 | Conduct problems | SB→MVPA | 0.004 | 0.011 | [-0.019, 0.026] | 0.754 |
| 15 | Conduct problems | LPA→MVPA | -0.011 | 0.015 | [-0.041, 0.019] | 0.472 |
| 15 | Conduct problems | SB→LPA | 0.014 | 0.007 | [0.001, 0.027] | 0.036* |
| 15 | Hyperactivity/inattention | SB→MVPA | 0.050 | 0.016 | [0.018, 0.082] | 0.002** |
| 15 | Hyperactivity/inattention | LPA→MVPA | 0.021 | 0.022 | [-0.022, 0.063] | 0.342 |
| 15 | Hyperactivity/inattention | SB→LPA | 0.029 | 0.010 | [0.010, 0.048] | 0.002** |
| 15 | Internalizing problems | SB→MVPA | -0.040 | 0.025 | [-0.089, 0.009] | 0.113 |
| 15 | Internalizing problems | LPA→MVPA | -0.062 | 0.033 | [-0.127, 0.003] | 0.061 |
| 15 | Internalizing problems | SB→LPA | 0.021 | 0.015 | [-0.007, 0.050] | 0.141 |
| 15 | Externalizing problems | SB→MVPA | 0.047 | 0.023 | [0.002, 0.092] | 0.041* |
| 15 | Externalizing problems | LPA→MVPA | 0.009 | 0.030 | [-0.050, 0.068] | 0.764 |
| 15 | Externalizing problems | SB→LPA | 0.037 | 0.013 | [0.010, 0.063] | 0.006** |
| 15 | SDQ total difficulties | SB→MVPA | 0.008 | 0.038 | [-0.067, 0.082] | 0.840 |
| 15 | SDQ total difficulties | LPA→MVPA | -0.046 | 0.051 | [-0.145, 0.053] | 0.362 |
| 15 | SDQ total difficulties | SB→LPA | 0.052 | 0.022 | [0.008, 0.096] | 0.020* |

*Note: Reference: geometric mean. All models adjusted for baseline SDQ at age 7, age, sex, ethnicity, family income, parental education, and K6. *p < 0.05, **p < 0.01, ***p < 0.001.*
